# Incidence and risk factors of peripheral nerve injuries 3 months after ICU discharge: a retrospective study comparing COVID-19 and non-COVID-19 critically ill survivors

**DOI:** 10.1186/s44158-024-00144-8

**Published:** 2024-02-09

**Authors:** C. Malengreaux, P. Minguet, C. Colson, N. Dardenne, B. Misset, A. F. Rousseau

**Affiliations:** 1 Department of Intensive Care and Burn Centre, University Hospital of Liège, Avenue de L’Hôpital,1, University of Liège, Sart-Tilman B35, Liège, B-4000 Belgium; 2 University and Hospital Biostatistics Centre (B-STAT), University of Liège, Liège, Belgium; 3 GIGA-Research, GIGA-I3 Thematic Unit, Inflammation and Enhanced Rehabilitation Laboratory (Intensive Care), University of Liège, Liège, Belgium

**Keywords:** Peripheral nerve injury, Critical illness, COVID-19, Glucocorticoids, Follow-up clinic

## Abstract

**Background:**

Peripheral nerve injuries (PNI) have been associated with prone positioning (PP) in mechanically ventilated (MV) patients with COVID-19 pneumonia. The aims of this retrospective study were to describe PNI prevalence 3 months (M3) after intensive care unit (ICU) discharge, whether patients survived COVID-19 or another critical illness, and to search for risk factors of PNI.

**Results:**

A total of 55 COVID (62 [54–69] years) and 22 non-COVID (61.5 [48–71.5] years) patients were followed at M3, after an ICU stay of respectively 15 [9–26.5] and 13.5 [10–19.8] days. PNI symptoms were reported by 23/55 (42.6%) COVID-19 and 8/22 (36%) non-COVID-19 patients (*p* = 0.798). As the incidence of PNI was similar in both groups, the entire population was used to determine risk factors. The MV duration predicted PNI occurrence (OR (CI95%) = 1.05 (1.01–1.10), *p* = 0.028), but not the ICU length of stay, glucocorticoids, or inflammation biomarkers.

**Conclusion:**

In the present cohort, PNI symptoms were reported in at least one-third of the ICU survivors, in similar proportion whether patients suffered from severe COVID-19 or not.

**Supplementary Information:**

The online version contains supplementary material available at 10.1186/s44158-024-00144-8.

## Background

Peripheral nerve injury (PNI) refers to damage to nerves located outside of the brain and spinal cord, mainly due to traumatic, ischemic, metabolic, infectious, or autoimmune causes [1]. Critically ill patients may develop PNI especially during prolonged stays in the intensive care unit (ICU) [2–4]. Ischemic hypoxemia, cytokines, oxidative stress, and stress hormones are known to induce primary distal axonal degeneration of motor and sensory fibers [5–7]. In particular, prolonged mechanical ventilation (MV) and prone positioning (PP) are described as situations at risk of PNI [3]: MV patients are more frequently subject to sepsis and have longer ICU length of stay (LOS) [8]. PNI commonly manifests as persistent pain, either hypo- or dysesthesia in a nerve territory. The diagnosis is mainly based on electrophysiological exams [9].

COVID-19 can lead to acute respiratory distress syndrome (ARDS) requiring ICU admission and MV for long periods [10]. An increasing number of neurological dysfunctions associated with this infection have been reported, such as encephalopathy, encephalitis, Guillain–Barré syndrome, and ischemic strokes [11]. In particular, SARS-CoV 2 could infect directly nerve cells, due to the affinity between the spikes on the viral surface and the angiotensin-converting enzyme 2 (ACE2) receptor [9, 12–15].

In June 2020, the RECOVERY study concluded to potential benefits of dexamethasone on the mortality of the COVID-19 patients receiving oxygen or mechanical ventilation [16]. Dexamethasone was then worldwide used as an adjuvant treatment for this disease. The role of glucocorticoids on peripheral nerves is controversial: their anti-inflammatory properties [17] could be counterbalanced by induced hyperglycemia [18, 19]. Their effects on PNI occurrence in COVID-19 ARDS survivors are unknown.

Up to now, it is unknown if survivors of critical COVID-19 suffer from PNI to a similar extent than survivors of other types of critical illness. The primary aim of the present retrospective study was to compare the PNI incidence in these two categories of ICU survivors. The second aim was to define the risk factors of PNI in both groups.

## Method

### Participants

Since 2019, patients surviving an ICU stay ≥ 7 days are routinely invited to our post-intensive care follow-up clinic, at 1, 3, and 12 months following ICU discharge. A multidisciplinary team, including critical care physicians, critical care nurses, physiotherapists, dieticians, and psychologists, is involved at each time. This face-to-face follow-up is standardized, addressing physical status and functional performances, nutritional status and body composition, bone health, mental health disorders, cognitive impairment, sleep disorders, and health-related quality of life (HRQoL). A blood analysis is also performed, focusing on inflammation and metabolic biomarkers.

In this retrospective study, we included all adults who attended the 3-month (M3) consultation at our follow-up clinic after a COVID-19 ARDS during the first wave (from March 1st to July 17th, 2020) and the second wave (from July 18th, 2020 to December 5th, 2020) of the pandemic. We also included patients who survived a non-COVID-19 critical illness between January 27th and July 27th, 2021 (non-COVID group). Patients were excluded from analysis in case of incomplete follow-up.

In accordance with Belgian law, informed consent was not required because the study did not modify patients’ management, and the data were anonymously collected. This interpretation was confirmed by the Ethics Committee of the University Hospital of Liege (local reference 2020/424).

### Procedures during COVID-19 pandemic

During the first wave of the pandemic, steroids were not used according to the Belgian public health service recommendations. The use of high-flow nasal oxygen (HNFO) was precocious (maximum flow of 30 L/min) due to the high potential risk of aerosolization. Prone position was performed in case of impaired oxygenation with PaO2/FiO2 ratio < 150 and/or FiO2 > 0.6. The duration of each prone positioning session was 16–18 h. Sessions were repeated at least three times if oxygenation improved. The proning procedure was performed according to the expert’s recommendations [20, 21], aiming at reducing the risk of accidental loss of invasive devices and pressure injuries. After appropriate preparation of the patient and the materials, proning was performed using a 4-step maneuver. The position of the head, arms, and legs was checked every 8 h by the nurses.

During the second wave, dexamethasone at the dose of 6 mg/day for 10 days was used in all patients who required oxygen therapy. The use of HNFO was more liberal (up to 60 L/min), and the criteria for the prone position were the same. Physiotherapists ensured mobilization in all patients with passive and active exercises when possible.

### Clinical variables

In all included patients, the same clinical variables were systematically collected during the M3 consultation: occurrence of PNI, level of autonomy for daily activities using the Barthel index, quality of life (QOL) using EQ-5D-3L scale PNI referred to limb weakness, pain, hypoesthesia or paresthesia. PNI incidence was based on clinical manifestation during the consultation with the physician at M3. There was no systematic complementary exam to confirm PNI. However, an electroneuromyography has been prescribed in some of the patients by physicians outside the post-ICU follow-up setting. The Barthel index of activities of daily living (ADL) was used to measure functional status and dependency. It consists of ten subheadings as feeding, bathing, grooming, dressing, bladder control, bowel control, toilet use, chair–bed transfer, mobility, and stair climbing [22]. Scoring ranges from 0 to 100, with a score of 100 defined as being capable of ADL complete self-care. HRQoL was measured using the EQ-5D-3L. This tool comprises two sections: a five-question descriptive component which explores five dimensions: mobility, self-care, usual activities, pain/discomfort, and anxiety/ depression. Each question has three possible answers, rated from 1 to 3: no problems, some problems, and extreme problems. The second section is a visual analog scale (EQ VAS) about HRQoL. Demographic data and data related to the ICU stay were also collected and extracted from the medical charts.

### Biological variables

The biological data were generated from one single laboratory (Unilab, CHU de Liège) accredited for ISO 15,189 Guideline. The following variables were collected: serum CRP (turbidimetric method, Alinity C), serum glucose (turbidimetric method, Alinity C), serum creatine kinase (turbidimetric method, Alinity C), glycated hemoglobin (capillary electrophorese, Sebia), and serum creatinine (turbidimetric method, Alinity C). The normal range is 0–5 mg/L for CRP, 70 mg/dL (3.9 mmol/L)–100 mg/dL (5.6 mmol/L) for glucose, < 5.7% for glycated hemoglobin, 0.55–1.02 mg/dL for creatinine in females and 0.55–1.18 mg/dL in males. The glomerular filtration rate (eGFR) was estimated using the MDRD equation during ICU stay and using creatinine-based CKD-EPI equations.

### Statistical analysis

Statistical analysis was performed using SAS (version 9.4 for Windows, SAS Institute, Cary, NC, USA) and R (version 4.0.2 for Windows) software.

The normality of the quantitative parameters was investigated using descriptive and graphical techniques (comparison of mean and median values, histogram, and quantile–quantile plot) and tested with the Shapiro–Wilk test. As some quantitative parameters were not normally distributed, results were expressed as medians with lower and upper quartiles (Q1–Q3), while qualitative parameters were summarized using the numbers (*n*) and frequencies (%).

The quantitative parameters were compared using the non-parametric Kruskal–Wallis test (KW) or ANOVA-1. The qualitative parameters were compared using the chi-squared test or Fisher’s exact test.

Variables that differed significantly between patients with and without PNI in the univariate analysis were included in a multivariate binary logistic regression analysis to identify those that remained independently associated with the occurrence of PNI. The same multivariate regression with a stepwise procedure was also computed. Using the Firth method, we estimated the odds ratios (OR) with 95% confidence interval (95% CI). The results were considered significant at the uncertainty level of *α* = 5% (*p* < 0.05).

## Results

### Patients

**Fig. 1:**
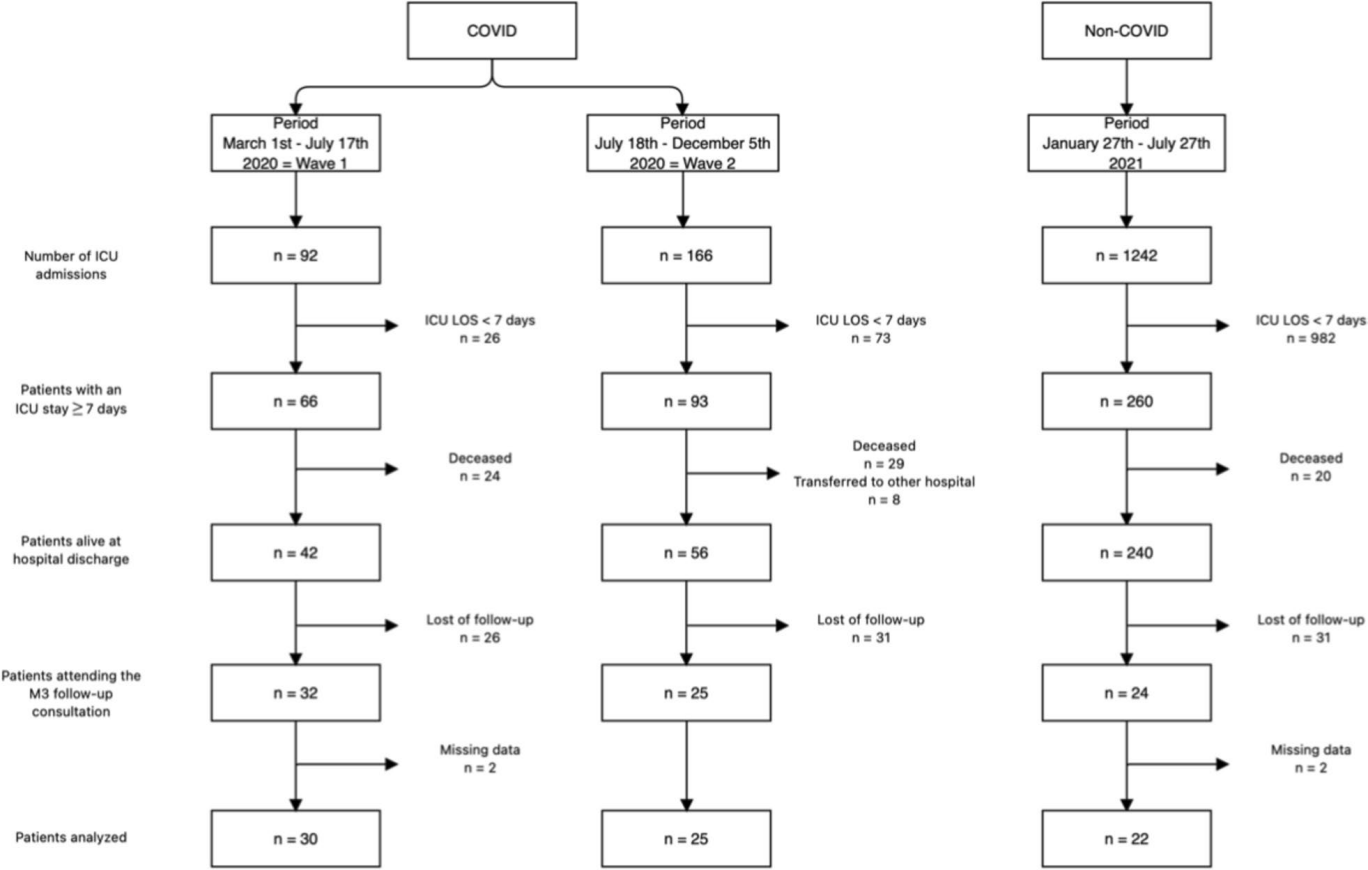
Flow chart

A total of 55 COVID patients and 22 non-COVID patients were included (Fig. 1). Their demographic parameters are detailed in Table 1. Demographic parameters did not differ significantly between the two groups. Hypertension or cardiovascular disease was more frequent in the COVID group, while active smoking was more frequent in the non-COVID group.

**Table 1:** Demographics and ICU data in the COVID and non-COVID groups

Data		COVID group (*n* = 55)	Non-COVID group (*n* = 22)	*p* value
Age (years)		62 [54–69]	61.5 [48–71.5]	0.99
Sex: male, *n* (%)		36 (65.5)	16 (72.7)	0.60
Weight, kg		92 [82–102]	79 [70–109]	0.06
BMI (kg/m^2^)		31.2 [28.7–33.9]	27.4 [24–38.8]	0.20
Medical history, *n* (%)	Chronic kidney disease	2 (3.6)	1 (4.5)	> 0.99
	Diabetes	26 (47.3)	8 (36.4)	0.38
	Hypertension	31 (56.4)	18 (81.8)	0.04
	Cardiovascular disease*	11 [20]	12 (54.5)	< 0.01
	COPD	11 [20]	3 (13.6)	0.74
	Asthma	5 (9.1)	0 (0)	0.32
	Immunosuppressive treatment	1 (1.8)	3 (13.6)	0.07
	Active smoking	4 (7.4)	8 (36.4)	< 0.01
Admission type, *n* (%)	Medical	55 (100)	9 (40.9)	< 0.01
Admission failure, *n* (%)	Cardiovascular	0	11 (50)	-
	Pulmonary	55 (100)	2 (9)	-
	Neurologic	0	6 (27)	-
	Other	0	3 (14)	-
SAPS II		33 [26–40]	34.5 [25–42]	0.97
Mechanical ventilation, *n* (%)		40 (75.5)	12 (54.5)	0.07
Duration of mechanical ventilation, days		17 [11–25]	6 [2–14]	< 0.01
Neuromuscular blocking duration, days		0 [0–4]	0 [0–0]	0.08
Prone position, *n* (%)		31 (56.4)	0 (0)	< 0.01
Corticosteroids, *n* (%)		40 (72.7)	3 (13.6)	< 0.01
Insulin infusion duration, days		0 [0–7]	0 [0–1.5]	0.44
CRP peak, mg/L		284 [189.4–338.7]	184.2 [149.4–260.8]	0.02
Glucose peak, mg/dL		229 [191–320]	215.5 [173.3–274.5]	0.22
ICU LOS, days		15 [9–27]	13.5 [10–20]	0.57
Hospital LOS, days		28 [20–48]	29.5 [20.2–42.2]	0.98

*BMI* body mass index, *CRP* C-reactive protein, *COPD* chronic obstructive pulmonary disease, *ICU* intensive care unit, *LOS* length of stay, *SAPS* simplified acute physiology score

^*^Cardiovascular disease, history of coronary bypass, percutaneous balloon angioplasty, or myocardial infarction

### PNI symptoms and their impact on the 3-month outcomes

Three months after ICU discharge, PNI symptoms were reported by 23/55 patients (42.6%) in the COVID group and by 8/22 (36%) patients in the non-COVID group (*p* = 0.798). The localizations of PNI were various, affecting both upper and lower extremities. The median, ulnar, radial, and sciatic nerves were the most frequently affected. An electroneuromyography was performed in 14/23 (60.8%) and in 1/8 (12.5%) patients of respectively the COVID and non-COVID groups. A pattern of axonal peripheric polyneuropathy was found in all these ENMGs.

Patients experiencing PNI symptoms had similar autonomy for ADL and estimated HRQoL compared to patients who did not report PNI symptoms (Table 2).

**Table 2:** Impact of PNI on 3-month outcomes

Parameters		PNI (*n* = 31)	No PNI (*n* = 46)	*p* value
Barthel		100 [100–100]	100 [100–100]	0.09
EQ-5D-3L	Total score (/15)	7 [6–8]	6.5 [6–8]	0.39
	VAS	66.87 [13.76]	69.14 [16.27]	0.53

*PNI* peripheral nerve injury, *VAS* visual analog scale

### PNI risk factors

As the incidence of PNI was similar in both groups, the entire population was used to determine risk factors. Among the recorded parameters, SAPS II, corticosteroids, prone position, insulin infusion duration, and glucose peak were not associated with the PNI occurrence (Table 3). On the contrary, the highest CRP blood concentration during the ICU stay, the duration of mechanical ventilation, and the ICU length of stay were significantly higher in the PNI group (Table 3). These three factors were included in the final multivariate binary logistic regression model. Only the duration of mechanical ventilation predicted PNI occurrence (OR (CI 95%), 1.05 (1.01–1.10, *p* = 0.028)).

**Table 3:** Studied clinical parameters in patients reporting PNI symptoms and in patients without PNI symptoms

Variables	PNI group (*n* = 31)	No PNI group (*n* = 46)	*p* value
SAPS II	33 [27–40]	33 [24–40.5]	0.76
Mechanical ventilation, *n* (%)	24 (77.4)	28 (63.6)	0.18
Duration of mechanical ventilation, days	15 [6.5–29]	6 [0–15]	< 0.01
Neuromuscular blocking duration, days	4 [3–5]	3 [2–5]	0.25
Prone position, *n* (%)	17 (60.7)	14 (37.8)	0.05
Corticosteroids, *n* (%)	17 (54.8)	26 (59.1)	0.71
Insulin infusion duration, days	11 [7–18]	14.5 [4.5–25]	0.71
CRP peak, mg/L	282 [219–353.3]	190.9 [127.3–309.1]	0.01
Glucose peak, mg/dL	220 [174–324]	230 [194.5–296]	0.82
ICU LOS, d	17 [12–35]	12 [8–21.5]	0.02

*CRP* C-reactive protein, *ICU* intensive care unit, *LOS* length of stay, *SAPS* simplified acute physiology score

### Subgroup analysis

The COVID group has been further divided into two subgroups, including either survivors of the first (*n* = 30) or the second wave (*n* = 25) of the pandemic. Demographics, clinical data, and outcomes assessment in the two subgroups are presented in Supplemental Table 1. Demographic characteristics were similar in the two subgroups. During the second wave, glucocorticoids were prescribed in all patients, who had shorter stays in the ICU with less prolonged vital supports. No difference in PNI incidence was observed between the first (14/30, 46.7%) and the second wave (10/25, 40%) (*p* = 0.789).

## Discussion

In the present retrospective study performed in a post-ICU follow-up clinic, PNI symptoms were reported by at least one-third of the patients 3 months after ICU discharge. This proportion was similar to whether patients survived a COVID-19 ARDS or another critical illness, refuting the notion that COVID and non-COVID ICU survivors should be followed differently, at least in terms of peripheral nerve injuries. These results are in line with previously reported prevalence. In non-COVID critically ill survivors, reported PNI based on electrophysiologic studies was observed in about 40% at ICU discharge [23–25]. In another cohort of patients who survived a COVID-19 requiring MV, 37.1% of the survivors reported symptoms of PNI 4 months after ICU discharge [13]. However, in an observational study in a small cohort during the first wave of the pandemic, PNI was observed in only 5% of critically ill survivors, diagnosed using ENMG. In these patients, muscle biopsy described scattered necrotic and regenerative fibers and non-specific lesions assuming that COVID-19-related PNI is no specific microscopic pattern [14].

In the present study, the duration of mechanical ventilation, but not the prone positioning, was found as a risk factor for PNI occurrence. A recently published systematic review including 41 studies analyzed the main adverse effects of prone positioning in critically ill patients with ARDS: PNI was observed in 8.1% of studied patients [26]. However, this finding could have been underestimated due to the lack of screening of PNI parameters during these studies.

The highest value of CRP during the ICU stay was the only biological parameter associated with PNI occurrence in the present univariate analysis. A similar finding was also reported in a retrospective study including critically ill patients who were diagnosed with PNI before the COVID-19 pandemic [8]. CRP blood concentration, a biomarker of systemic inflammation, has been associated with a decrease of the peripheral nerve action potential amplitude in critically ill patients with SIRS: a negative correlation was found between CRP level and compound muscle action potential amplitude [8]. Glucocorticoids, with their anti-inflammatory effects, could theoretically reduce PNI incidence in a critical care context [17]. Such a positive effect has not been observed in our present study, nor in other studies in critically ill patients. A prospective, double-blind randomized study on persistent ARDS patients in whom glucocorticoids vs placebo were administrated did not lead to a reduction in PNI prevalence [27]. Moreover, glucocorticoids have even been incriminated in the pathophysiology of myopathy that include fiber atrophy and myosinolysis due to stimulation of corticosteroid muscle receptors [18]. However, the use of glucocorticoids in the context of high inflammatory status can be a confounding factor. Altogether, studies aiming to define risk factors of PNI in critically ill patients did not identify glucocorticoids as one of them [28–30].

Long-term outcomes of patients with PNI are now increasingly evaluated. A complete recovery is reported in 50 to 75% of the patients who survived critical illness with PNI one year after ICU discharge [7, 31]. For those who still experience PNI at 1-year follow-up consultation, their quality of life could still be significantly altered with poor physical stress tolerance and easy fatigability [32]. In the present study, the quality of life and the autonomy for ADL 3 months after ICU discharge were similar, whether patients presented symptoms of PNI or not.

Some limitations need to be acknowledged. First, the size of the studied population was limited and the study may be underpowered. Further investigations will be required to confirm the present findings. Second, some patients had clinical diagnoses of PNI without confirmation with ENMG. In some cases, the diagnosis was performed by a non-neurologist. The number of PNI could thus have been either overestimated or underestimated. However, similar incidences of PNI were observed in studies in which PNI was diagnosed with an EMG [13, 23, 25]. Finally, a number of patients were lost to follow-up, partly due to reduced human resources in our follow-up clinic during the COVID pandemic. Some patients refused to attend the consultation: they could have been either patients without any complaints or, in contrast, bedridden patients. However, these two categories of survivors are probably those who would benefit the least from a follow-up clinic. Altogether, these issues could have led to an unwanted selection bias. This bias is unfortunately inherent in follow-up studies, as observed in previously published cohorts.

## Conclusion

In the present cohort retrospectively assessed 3 months after a prolonged ICU stay, PNI symptoms were reported in at least one-third of the survivors, in similar proportion whether they suffered from severe COVID-19 or not. PNI symptoms did not seem to impact autonomy or health-related quality of life. Only the duration of mechanical ventilation was found to be a PNI risk factor. A better understanding of inflammation as a PNI driver should be the goal of future research.

### Supplementary Information

**Additional file 1: Supplemental Table 1. **Demographic, clinical and outcome data in patients admitted during the two first waves of the pandemics.

## Data Availability

The datasets used and/or analyzed during the current study are available from the corresponding author on reasonable request.

## Abbreviations

ADL: Activities of daily living
ARDS: Acute respiratory distress syndrome
BMI: Body mass index
ENMG: Electromyoneurography
HFNO: High-flow nasal oxygen
HRQoL: Health-related quality of life
ICU: Intensive care unit
LOS: Length of stay
MV: Mechanical ventilation
PNI: Peripheral nerve injury
PP: Prone positioning
SIRS: Systemic inflammatory response syndrome
VAS: Visual analog scale
