# Supplementary material for: Incidence and risk factors of peripheral nerve injuries 3 months after ICU discharge: a retrospective study comparing COVID-19 and non-COVID-19 critically ill survivors
Source: J Anesth Analg Crit Care. 2024 Feb 9;4:10. doi: 10.1186/s44158-024-00144-8 (PMC10858596; doi:10.1186/s44158-024-00144-8)
Supplement: Supplementary file 1 — Additional file 1: Supplemental Table 1. Demographic, clinical and outcome data in patients admitted during the two first waves of the pandemics. [file 44158_2024_144_MOESM1_ESM.docx]

**Incidence and risk factors of peripheral nerve injuries 3 months after ICU discharge: a restrospective study comparing COVID-19 and non-COVID-19 critically ill survivors.**

C. Malengreaux, MD^1^; P. Minguet, RN^1^; C. Colson, RN^1^; N. Dardenne, MSc ^2^;

B. Misset, MD^1^; A.F. Rousseau, MD, PhD^1,3^

Supplemental Table 1: Demographic, clinical and outcome data in patients admitted during the two first waves of the pandemics.

| Data |  | Wave 1 (n = 30) | Wave 2 (n = 25) | p value |
| --- | --- | --- | --- | --- |
| Age, years | | 62 [50-68] | 62 [55.5-71] | 0.20 |
| Males, n (%) | | 21 (70) | 16 (64) | 0.78 |
| Weight, kg | | 96 [85-105] | 87 [77.5-100] | 0.09 |
| BMI, kg/m^2^ | | 31.2 [29.8-33.9] | 31.5 [26.5-34.5] | 0.55 |
| Medical history, n (%) | Chronic kidney disease | 2 (6.5) | 0 (0) | 0.50 |
|  | Diabetes | 16 (53.3) | 10 (40) | 0.43 |
|  | Hypertension | 16 (53.3) | 15 (60) | 0.60 |
|  | Cardiovascular disease* | 7 (23.3) | 4 (16) | 0.74 |
|  | COPD | 3 (10) | 8 (32) | 0.05 |
|  | Asthma | 3 (10) | 2 (8) | >0.99 |
|  | Immunosuppressive treatment | 1 (3.3) | 0 (0) | >0.99 |
|  | Active smoking | 3 (10) | 2 (8) | >0.99 |
| SAPS II | | 36.5 [29-43.7] | 29 [24-33.5] | <0.01 |
| Mechanical ventilation, n (%) | | 27 (90) | 13 (52) | <0.01 |
| Duration of mechanical ventilation, d | | 21 [12-27] | 10 [5.7-16.5] | 0.01 |
| Neuromuscular blocking duration, d | | 3.5 [0-4.7] | 5.5 [4.2-11.2] | <0.01 |
| Prone position, n (%) | | 24 (80) | 8 (32) | <0.01 |
| Corticosteroids, n (%) | | 15 (50) | 25 (100) | <0.01 |
| Insulin infusion duration, d | | 3.5 [0-22.8] | 0 [0-0] | <0.01 |
| CRP peak, mg/L | | 298.2 [245.4-360.1] | 218.1 [159.9-310.1] | 0.02 |
| Glucose peak, mg/dL | | 218 [173.5-297] | 240.5 [197-332.5] | 0.36 |
| ICU LOS, d | | 23.5 [14.7-40.5] | 9 [6.5-15] | <0.01 |
| Hospital LOS, d | | 40 [27.7-57] | 22 [12.5-27.5] | <0.01 |
| PNI incidence, n (%) | | 14 (46.7) | 10 (40) | 0.79 |

BMI: body mass index; COPD: chronic obstructive pulmonary disease; CRP: C-Reactive protein; ICU: intensive care unit; LOS: length of stay; SAPS: Simplified Acute Physiology Score

*Cardiovascular disease = History of coronary bypass, percutaneous balloon angioplasty or myocardial infarction
